# Novel Rim Plating Technique for Treatment of the Inferior Pole Fracture of the Patella

**DOI:** 10.1111/os.12876

**Published:** 2021-02-22

**Authors:** Qi‐fang He, Guo‐biao Pan, Ze‐feng Yu, Wang‐xiang Yao, Liu‐long Zhu, Cong‐feng Luo, Xiao‐shan Guo

**Affiliations:** ^1^ Department of Orthopaedic Surgery Affiliated Hangzhou First People's Hospital Zhejiang University School of Medicine Hangzhou China; ^2^ Department of Orthopaedic Surgery Hangzhou Cancer Hospital Hangzhou China; ^3^ Department of Orthopaedic Surgery Shanghai Jiao Tong University Affiliated Sixth People's Hospital Shanghai China; ^4^ Department of Orthopaedic Surgery The Second Affiliated Hospital of Wenzhou Medical University Wenzhou China

**Keywords:** Inferior patellar fracture, Internal fixation, Rim plating

## Abstract

To aim of the present paper was to introduce a novel fixation technique for the treatment of inferior pole fracture of the patella. We performed a prospective observational study of consecutive cases of inferior pole fracture of the patella that were treated at our institution between January 2018 and June 2019. The patients include three men and one woman, with an average age of 47 years (range: 42–59 years). All patients were treated with the novel rim plating fixation technique for preserving the inferior pole of the patella. During the surgery, a 2.4 mm straight locking compression plate was contoured to adapt to the arc of the lower half of the patella as the rim plate. After reduction of the fracture, the rim plate was fixed to the proximal fragment of the patella through multiple locking screws, against the continuous pull of the patellar tendon. The rim plate encircles and constricts the inferior pole fragments, functioning as a compression and blocking construct. If necessary, an additional anterior tension band or mini locking plate can be used to further prevent anterior displacement of the inferior pole fragments. Under this rigid fixation, motion of the knee and full weight‐bearing were encouraged postoperatively. The patients were followed up monthly until 12 months after surgery. The time to achieve 90°pain‐free, full range of motion of the knee, and fracture healing, were recorded. Related complications were monitored, including infection, loss of reduction, fixation failure, anterior knee pain, and soft‐tissue irritation. The modified Cincinnati knee rating system was used for knee function assessment. The average operative time was 58.8 min (range: 52–63 min). The average blood loss was 59.8 mL (range: 45–71 mL). For all patients, pain‐free 90° range of motion was restored in 2–4 weeks, and the full range of motion was restored in 8–11 weeks. All patients achieved bone union in 6–9 weeks with no displacement of the fragments or breakage of the implant. No patient complained of anterior knee pain or soft‐tissue irritation. The modified Cincinnati score at 12‐month follow up demonstrated excellent outcomes in all four patients. The rim plating technique may be a feasible option for the treatment of the inferior pole fracture of the patella.

## Graphical abstract

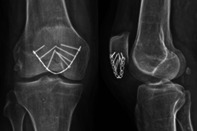

During the surgical treatment of the inferior pole fracture of the patella, the rim plating technique can prevent postoperative displacement of the inferior pole fragment and enable early mobilization of the injured knee.

## Introduction

The patella is a triangular bone situated on the anterior surface of the knee at the distal end of the femur. It is the largest sesamoid bone in the human body and forms part of the knee joint. The patella plays a crucial role in the transmission of the quadriceps muscle forces, the increase of the lever arm, the distribution of the forces on the femoral trochlea, and the centering of the extensor apparatus^1^. The inferior pole of the patella is an anterior bony extension of the patella body, which is the origin of the patellar tendon and bears high stress. Fractures of the patella comprise 1% of all fractures, with an incidence of approximately 1.2 per 100,000 per year^2^. Fractures of the patella may result from either direct or indirect mechanisms. The indirect eccentric tension through the extensor mechanism often results in avulsion fractures of the inferior pole, causing fracture displacement. Among all patellar fractures that require surgical treatment, fractures of the inferior pole of the patella account for 9.3% to 22.4%^3^. These patellar fractures directly interrupt the knee extension structure and are seldom stable. To restore the continuity of the knee extensor and allow early knee motion, surgical treatment is often necessary for the management of this group of fractures, while nonoperative management is reserved for a small proportion of non‐displaced and stable fractures^4^.

Thus far, the two main surgical options available are (i) partial patellectomy, entailing resection of the inferior pole fragment and reconstruction of the extensor using transosseous sutures or wires; and (ii) osteosynthesis, involving preserving the inferior pole of the patella using various fixation techniques described in the published literature^5^. Due to the difficulty of fixation to the small inferior pole fragments, partial patellectomy and repair of the patellar tendon with sutures was once the treatment of choice^6^, ^7^. However, the connection of tendon to bone is relatively weak and takes a longer time to heal than a bone‐to‐bone interface, which often requires the patients to be in prolonged immobilization before knee mobilization resumes. In addition, resection of the inferior pole of the patella is often associated with complications, including postoperative alteration in the length of the patella (patella baja), anterior knee pain, stiff knee, and decrease of quadriceps power^6^, ^8^. Under such circumstances, most surgeons prefer to preserve the inferior pole fragments through internal fixation^9^. However, under the continuous stress from the patellar tendon, it is hard to achieve a fixation rigid enough to control these small‐sized fragments. The difficulty may be further exacerbated by multiple risk factors, such as a multifragmentary fracture and poor bone quality of elderly patients^5^, ^10^. An ideal fixation method should comply with three crucial demands: it should aid in reduction, provide stable fixation, and enable early rehabilitation^11^. Such a method has not yet been identified. The methods of traditional tension band and cerclage wiring are not useful as they do not provide satisfactory stability if the distal pole is comminuted and are often associated with loss of reduction and soft tissue irritation due to breakage and migration of the wiring^4^. Therefore, various new techniques have been described in the literature for osteosynthesis of the inferior pole of the patella, with the primary goal of reliable fixation^12^, ^13^, ^14^, ^15^, ^16^. However, most of the current techniques remain less helpful for comminuted inferior pole fractures and are commonly associated with certain complications.

Over the past decade, the popularity of the locking plate in treating patellar fractures has been increasing. Some anatomical locking plates are used to manage comminuted patellar fractures, offering high coverage and stable locking fixation^5^, ^17^. In addition, specific plate constructs have been developed to manage inferior pole fractures of the patella, such as the basket plate, the hook plate, and the button plate^8^, ^13^, ^14^. However, to our knowledge, there is no current technique using a rim plate beneath the patellar tendon to fix the inferior pole of the patella. We present a novel technique of rim plating, which may be an effective and safe option for the treatment of these challenging fracture patterns. The purpose of this study is: (i) to assess the feasibility of the rim plating technique in treating inferior pole fractures of the patella; (ii) to confirm the efficacy of the technique; and (iii) to evaluate the safety of the surgery.

## Surgical Technique

### *Inclusion and Exclusion Criteria*

The inclusion criteria were as follows: (i) patients diagnosed with type 34‐A1 patellar fractures; (ii) patients treated operatively and fixed with the rim plating technique; (iii) postoperative follow up not less than 1 year; (iv) full postoperative radiological and clinical outcomes were acquired; and (v) a prospective study. The exclusion criteria were: (i) pathological or non‐traumatic fracture; (ii) open fractures; (iii) patients with prior limited knee function; and (iv) patients who refused to undergo rim plating fixation.

### *Concept and Mechanism of the Rim Plating Technique*

**Fig. 1:**
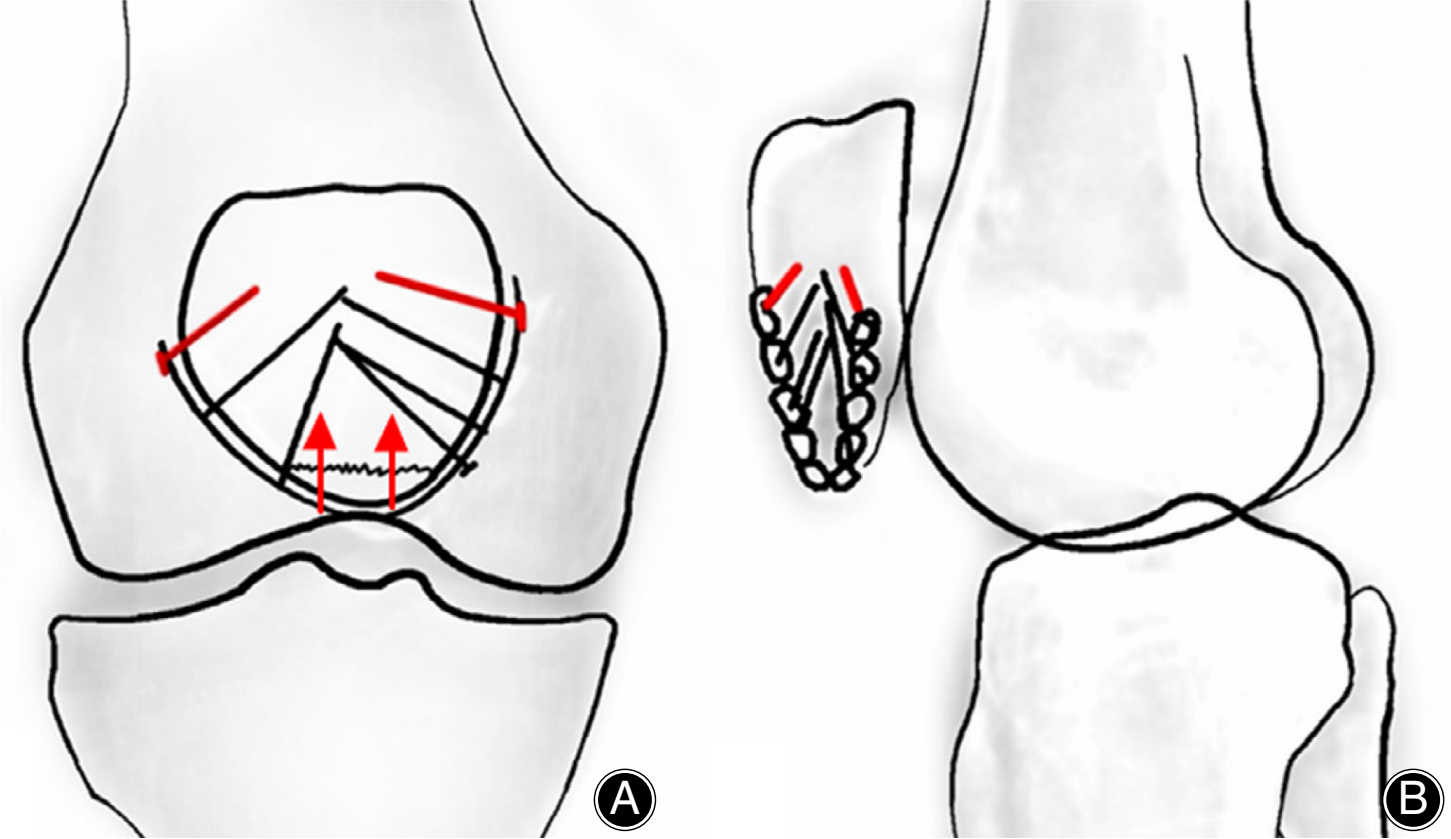
Diagram of the rim plating technique in anterior–posterior (A) and lateral view (B). The two cortical screws (marked red) are initially implanted. During the tightening of the two cortical screws, the distal fragment was pulled proximally (red arrow). Then the locking screws (black) were implanted.

The rim plate encircling the inferior pole of the patella would function as a compression and blocking construct. In treating a simple fracture with an intact inferior pole fragment, following the appropriate implantation of the two proximal cortical screws, the plate would slightly migrate proximally. This migration of the plate would push the inferior fragment towards the proximal patella, creating a preload between the fragments. This static compression, although different from the dynamic compression produced by the tension band, would also facilitate bone healing. Simultaneously, the lower arc part of the plate body nestles the displaced inferior pole fragments into their original location and blocks them from distal displacement. The blocking effect created by this “plate dam” is firm and reliable. Especially in dealing with comminuted fractures, all fragments would be constructed as a whole, preventing the separation between distal fragments. The strength of this fixation relies on the rigidity of the plate and the locking screws fixed to the side edges of the proximal patella. Multiple screws bear the stress through sufficient anchorage, which would largely avoid cutting through the bone (Fig. 1).

### *Operative Procedure*

Under spinal or general anesthesia, the patient was placed in the supine position on the radiolucent table. The injured leg was draped in a manner that would allow free movement during the operation. After the inflation of a pneumatic tourniquet, the knee was positioned in extension.

A straight midline incision, approximately 8 cm in length, was made from the upper 1/3 of the body of the patella to the inferior pole of the patella. After skin incision, full‐thickness soft‐tissue flaps were raised medially and laterally to expose the fragments, the ruptured retinaculum, and the patellar tendon. Elevation of the periosteum around the fragments is unnecessary. The blood clots and debris were removed and the knee joint was irrigated through the fracture gap.

**Fig. 2:**
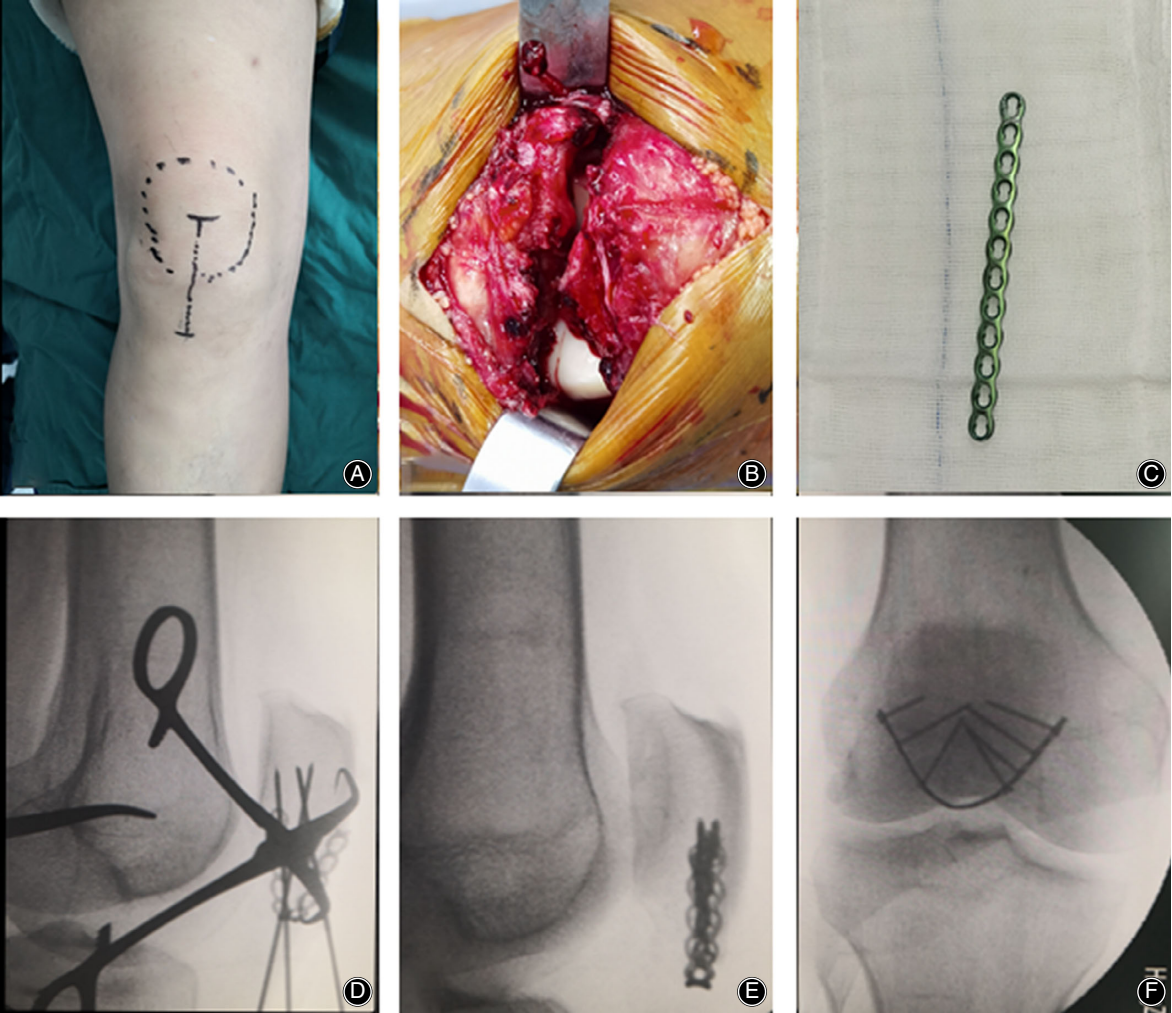
Case 1: A 56‐year‐old woman sustained an inferior patellar pole fracture of the right knee from a scooter accident. (A) The straight midline incision. (B) The fracture and knee joint were exposed and irrigated. (C) A 2.4 straight locking plate with sufficient length was prepared. (D) The fracture was reduced in knee extension and maintained by a towel laying forceps. (E and F) The plate was contoured to encircle the inferior pole fragment. The fracture realignment and rim plating were confirmed by intraoperative fluoroscopy.

**Fig. 3:**
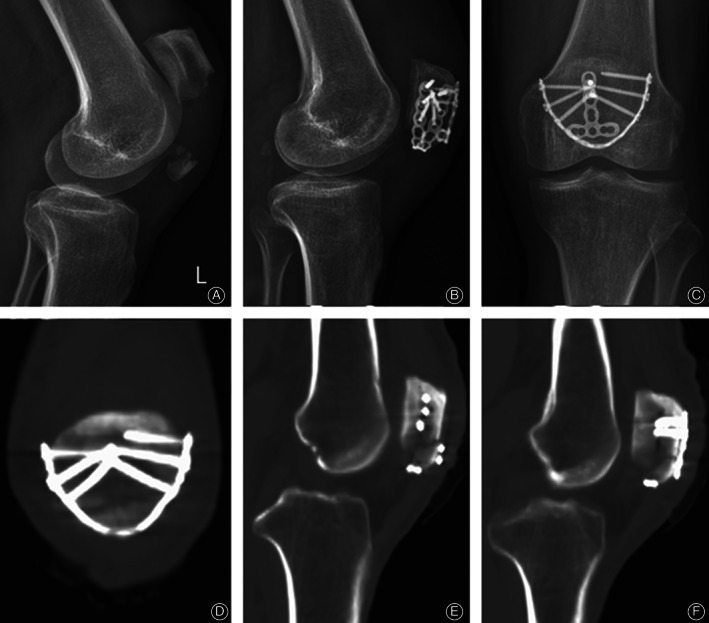
Case 2: A 48‐year‐old man suffered from an inferior patellar pole fracture of the left knee. (A) Obvious displacement of the comminuted inferior pole of the patella. (B, C) The fracture was reduced and fixed with a rim plate, with an anterior mini 2.0 locking plate to prevent anterior displacement of the distal fragments. (D–F) Postoperative CT display: the distal fragments were wrapped by the rim plate on both coronal and sagittal planes. The insertion of the patellar tendon was free from the implants.

A 2.4 mm straight locking compression plate was prepared and contoured to adapt to the arc of the lower half of the patella. Forceps were used to reduce the fracture and maintain the reduction temporarily. The anatomic reduction was confirmed by intraoperative fluoroscopy. At the rupture of the retinaculum, the curved plate is horizontally inserted to hold the inferior pole fragment close to the proximal fragment. The key point is to locate the locking plate as close as possible to the insertion of the patellar tendon. Two cortical screws were implanted at the bilateral ends of the plate for compression when upward traction was applied to the plate using two forceps. After confirmation of the position of the plate by intraoperative fluoroscopy, locking screws were fixed to hold the proximal fragment and stabilize the plate (Fig. 2). After the completion of rim plating, an anterior tension band was made with PDS‐II sutures to prevent anterior displacement of the inferior pole. If the distal fragments are too comminuted to be constricted by the anterior tension band, an anterior mini buttress plate is recommended (Fig. 3). When fixation was complete, immediate passive motion was used to ensure the stability before the closure of the incision.

### *Rehabilitation*

Motion of the knee and full weight‐bearing were encouraged postoperatively. In the first 2 weeks after the operation, the knee was unrestricted, but activities with intensity or flexion of more than 90° were not recommended.

## Outcome Measures

### *Operative Time and Blood Loss*

The operative time was recorded for a general assessment of the difficulty and complexity of the surgical procedure. The degree of surgical trauma was evaluated by intraoperative blood loss.

### *Visual Analog Scale*

The visual analog scale (VAS) is a validated, subjective measure for acute and chronic pain. Scores are recorded by making a handwritten mark on a 10‐cm line that represents a continuum between “no pain” and “worst pain.” The VAS was used to evaluate the postoperative knee pain after surgery.

### *Fracture Healing Time*

The most common clinical criteria of fracture healing are the absence of pain or tenderness during weight‐bearing, the absence of pain or tenderness on palpation or physical examination, and the ability to bear weight. The time of the fracture meeting the clinical healing criteria is considered the fracture healing time. A prolonged fracture healing time often indicates an unstable fixation or impairment to the blood supply.

### *Loss of Reduction*

Loss of reduction is defined as the inferior pole fragment being displaced more than 2 mm in any direction, which is assessed by observation of radiographic images. A significant loss of reduction indicates the failure of fixation.

### *Time to Achieve 90° Pain‐Free and Full Range of Motion*

The range of motion (ROM) is the measurement of the amount of movement of a specific joint, which can be measured using a goniometer. Prolonged immobilization would cause a decrease in ROM. The period to a full ROM is associated with the time and effect of postoperative rehabilitation.

### *Modified Cincinnati Score*

The Modified Cincinnati Rating System Questionnaire rating system consists of 12 questions, covering the domains of pain, swelling, function, and activity level. The total score is calculated as the sum of all questions' responses, with 100 representing the best/excellent knee function and 0 representing the worst/poor knee function. This system can help to evaluate the change following surgery or other interventions to the knee.

### *Complications*

The potential complications, as an indicator of the safety of the surgery, include infection, internal fixation failure or breakage, anterior knee pain, knee stiffness, and patella baja. All complications were recorded. The incidence of complications influences the safety and feasibility of the surgery.

### *Statistical Analysis*

All the calculations were achieved using IBM SPSS Statistics version 25 (IBM SPSS Statistics for Windows 64 bit, version 22.0; IBM, Armonk, NY, USA), with significance for all analyses set at *P* < 0.05. The estimates from normally distributed data are reported as means and ranges.

## Results

Between January 2018 and June 2019, four consecutive patients with isolated inferior patellar fractures were treated with the rim plating technique in a level I trauma center. The patients included three men and one woman. One female patient was diagnosed with osteoporosis.

### *Follow‐Up*

The follow‐up period was 12 months after primary surgery. All patients were usually followed up in routine outpatient clinics, first at 1 month, then 3‐monthly until the 12th month. An Internet video call would be arranged if the patient could not come to an outpatient clinic as scheduled. During each follow up, a physical examination was performed, and the ROM of the involved knee was measured. A routine X‐ray was taken to evaluate the fracture displacement and conditions of the implants. The patients were asked to fill in The Modified Cincinnati Rating System Questionnaire at 12 months postoperation. The details of the outcomes are described in Table 1.

**TABLE 1:** Patients' demographic information and outcomes

Patients (number)	1	2	3	4
Gender (F/M)	F	M	M	M
Age (years)	56	48	59	42
Operation duration (min)	59	61	63	52
Blood loss (mL)	65	45	58	71
Fracture healing time (weeks)	9	6	8	8
Time to 90° range of motion (weeks)	4	3	3	2
Time to full range of motion (weeks)	11	9	9	8
Modified Cincinnati score*	89	93	91	97

* Evaluated 12 months postoperation.

### *General Results*

The operations were performed routinely 2 days after hospitalization. The average operative time was 58.8 min (range: 52–63 min). The average blood loss was 59.8 mL (range: 45–71 mL). Two patients received Celebrex 400 mg per day for 3 days after the operation. Each patient was discharged from the hospital around 1 week after the operation.

### *Clinical Improvement*

All four patients could walk independently with weight‐bearing within 1 week. The average VAS at 1 month after the operation was 1.5 (range: 0–3). The clinical bone union was observed between 6 and 9 weeks.

### *Radiographic Evaluation*

**Fig. 4:**
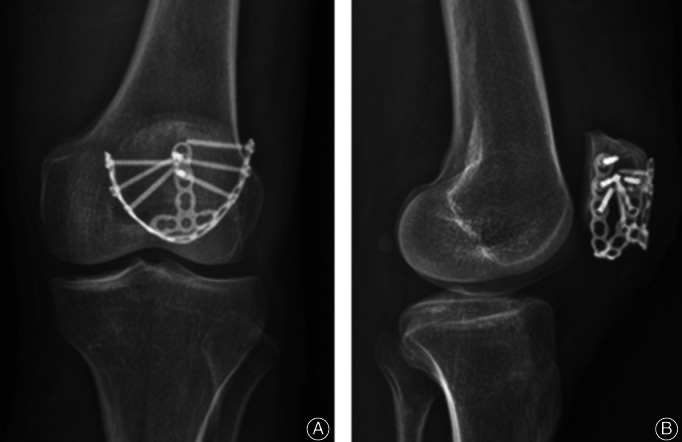
Case 2: At 12‐month follow‐up, fixation failure or patella baja was not noted (A, B).

At 12‐month follow up, no displacement of the fragments or breakage of the implants was noted. No patella baja was present on radiographic imaging (Fig. 4).

### *Functional Outcomes*

**Fig. 5:**
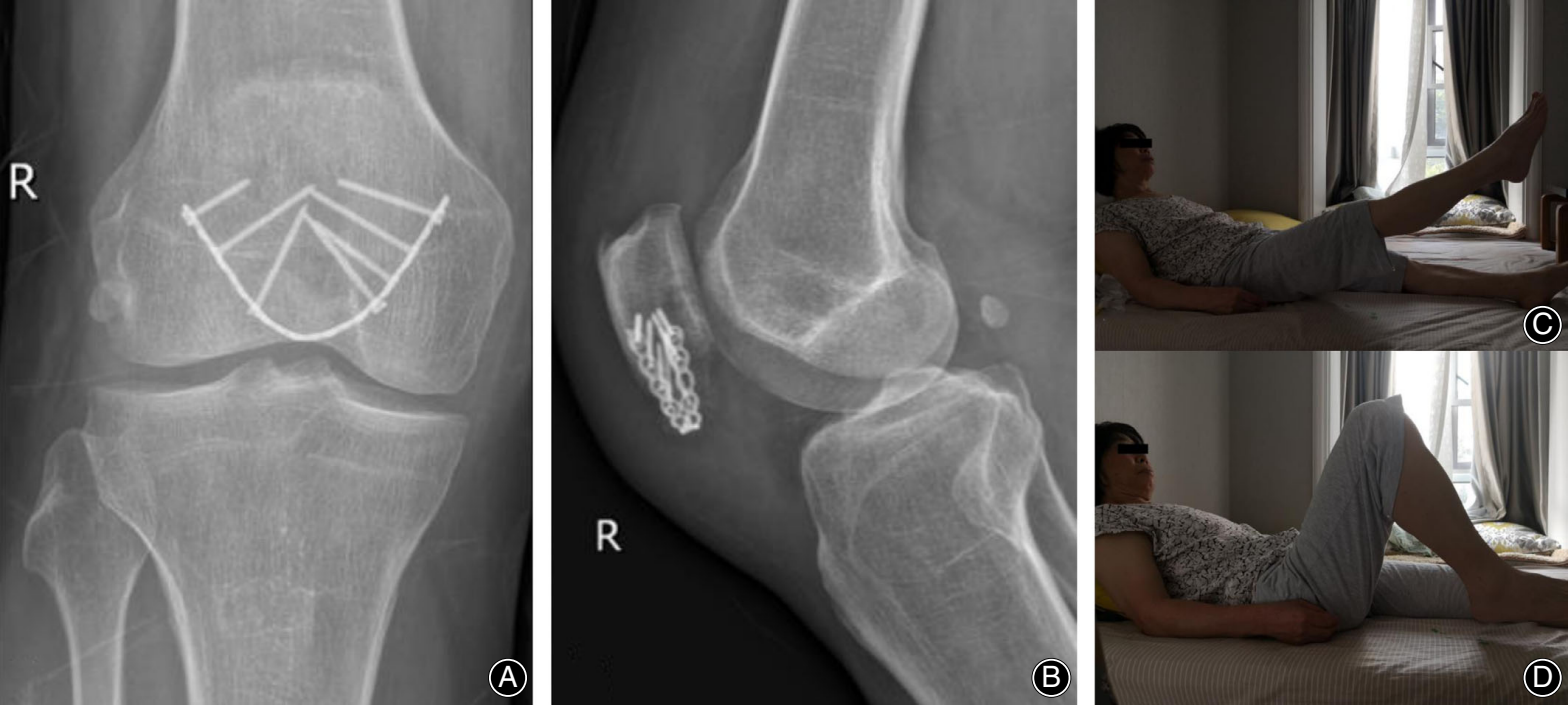
Case 1: At 11 weeks after the operation, the fracture healed without loss of reduction and the implant remained intact (A, B). Full range of motion was achieved (C, D).

The 90° pain‐free ROM was restored in 2–4 weeks, and the full ROM was restored in 8–11 weeks (Fig. 5). The modified Cincinnati score at 12‐month follow‐up ranged from 89 to 97.

### *Complications*

No infection was observed. No patient complained about anterior knee pain or soft‐tissue irritation and no patient required implant removal.

## Discussion

### *Current Status*

To our knowledge, no consensus has been reached about the ideal method for fixing inferior pole fractures of the patella. The inherent weakness and the size of the fragments rule out firm stabilization with commonly used wiring or screws. In recent published literature, basket plate fixation and separate vertical wiring are regarded as the two most reliable techniques^18^. Biomechanically, the basket plate can sustain a peak force of more than 400 N from the patellar tendon^19^. The basket plate fixation allows early unrestricted motion of the knee and its efficacy has been confirmed by long‐term studies^11^. However, there are some concerns about the large size of the plate and its location directly at the patellar tendon, from which irritation of the soft tissues could arise^17^. In addition, the basket plate is not available in many countries and most orthopaedic surgeons are inexperienced in using this plate. Comparatively, the separate vertical wiring is more cost‐effective, with easily obtained materials. The ultimate load of this fixation system is 250.1 ± 109.7 N, and the force loaded to the completely extended quadriceps muscle is 316 N; therefore, 4 weeks of long leg cast immobilization of the injured knee is needed^16^. Song *et al*. (2014) augmented this technique with cerclage wire, achieving a higher average ultimate failure load of 325 N, but a sudden contracture of the quadriceps is still a threat to the fixation^20^. The main limitation of the separate vertical wiring is that its efficacy in the treatment of comminuted fractures in the elderly remains uncertain.

### *Feasibility of the Novel Technique*

This is the first study attempting to fix the inferior pole of the patella with a single rim plate from below the patellar tendon. Anatomically, the patellar tendon extends to the quadriceps tendon through the surface of the patella. It is virtually impossible to restrict the pole fragments completely from the surface of the patella without interference to the patellar tendon. Among current forms of fixation, the implants either thrust through the patellar tendon or compress on its surface^11^, ^13^, ^14^, ^19^, ^21^. The soft tissue irritation waiting to happen necessitates a second surgery for implant removal. In fact, it is feasible to place a mini locking plate beneath the patellar tendon and along the inferior pole coronally^10^. Placing the plate parallel to the patellar tendon would theoretically minimize the disturbance to the tendon and surrounding soft tissue. For this reason, the 2.4 mm locking compression plate was selected for this special position, as it is neither bulky nor too weak to bear the stress. This hypothesis is preliminarily confirmed by the outcomes. The rim plate did not cause laceration or irritation of the patellar tendon, and no patient complained about the prominence of the implant.

### *Efficacy and Safety*

In terms of the mechanism, the rim plate works as a blocking construct, rather than a gripping structure. The cerclage wiring or the basket or hook plate holds the inferior pole fragments through longitudinal gripping force from the wires or hooks, while the rim plate blocks the fragments directly with part of the plate around the pole. This blocking mechanism with evenly distributed resistance may achieve better control of the inferior pole fragments, and potentially reveal the advantage in circumstances of multifragmentary fractures or poor bone quality. With this coronal plating, the pull strength from the patellar tendon is confronted by the purchase of multiple locking screws, so the cerclage around the patella to enhance the fixation is no longer necessary. In the intraoperative test, after plating, the inferior pole fragments were rigidly stabilized with a range of motion between 0° and 120°. The inferior pole fragment inclined to displace anteriorly when the knee was put in extreme flexion. To eliminate this, we suggest placing an anterior tension band or mini anti‐glide plate to assist after the rim plating. We suggest not restricting the knee after the operation, which may help in regaining the muscle strength quickly and reduce the risk of deep venous thrombosis. However, activities with intensity or high flexion of the knee are not recommended. The pursuit of full ROM prematurely is not advisable.

The novel technique has limitations. First, we have not performed a biomechanical test for this technique yet. Although the intraoperative test demonstrated a stable fixation, a biomechanical evaluation is necessary before further clinical use of the technique. Second, only four patients have received the fixation with the rim plating technique. The excellent outcomes may not be representative when larger samples are involved. Because the overall incidence of this type of fracture is low, a multicenter trial may be needed for further evaluation of the technique.
